# Watershed analysis of urban stormwater contaminant 6PPD-Quinone hotspots and stream concentrations using a process-based ecohydrological model

**DOI:** 10.3389/fenvs.2024.1364673

**Published:** 2024-03-06

**Authors:** Jonathan J. Halama, Robert B. McKane, Bradley L. Barnhart, Paul P. Pettus, Allen F. Brookes, Angela K. Adams, Catherine K. Gockel, Kevin S. Djang, Vivian Phan, Sonali M. Chokshi, James J. Graham, Zhenyu Tian, Katherine T. Peter, Edward P. Kolodziej

**Affiliations:** 1 U.S. Environmental Protection Agency, Corvallis, OR, United States,; 2 Independent Researcher, Middleton, WI, United States,; 3 U.S. Environmental Protection Agency, Seattle, WA, United States,; 4 Inoventures Inc, Corvallis, OR, United States,; 5 Cal Poly Humboldt, Arcata, CA, United States,; 6 Northeastern University, Boston, MA, United States,; 7 Center for Urban Waters, Tacoma, WA, United States,; 8 University of Washington Tacoma, Tacoma, WA, United States,; 9 University of Washington, Seattle, WA, United States

**Keywords:** VELMA, 6PPD-quinone, fate and transport, stormwater, modeling, TRWP, urban contaminant, coho

## Abstract

Coho salmon (*Oncorhynchus kisutch*) are highly sensitive to 6PPD-Quinone (6PPD-Q). Details of the hydrological and biogeochemical processes controlling spatial and temporal dynamics of 6PPD-Q fate and transport from points of deposition to receiving waters (e.g., streams, estuaries) are poorly understood. To understand the fate and transport of 6PPD and mechanisms leading to salmon mortality Visualizing Ecosystem Land Management Assessments (VELMA), an ecohydrological model developed by US Environmental Protection Agency (EPA), was enhanced to better understand and inform stormwater management planning by municipal, state, and federal partners seeking to reduce stormwater contaminant loads in urban streams draining to the Puget Sound National Estuary. This work focuses on the 5.5 km2 Longfellow Creek upper watershed (Seattle, Washington, United States), which has long exhibited high rates of acute urban runoff mortality syndrome in coho salmon. We present VELMA model results to elucidate these processes for the Longfellow Creek watershed across multiple scales–from 5-m grid cells to the entire watershed. Our results highlight hydrological and biogeochemical controls on 6PPD-Q flow paths, and hotspots within the watershed and its stormwater infrastructure, that ultimately impact contaminant transport to Longfellow Creek and Puget Sound. Simulated daily average 6PPD-Q and available observed 6PPD-Q peak in-stream grab sample concentrations (ng/L) corresponds within plus or minus 10 ng/L. Most importantly, VELMA’s high-resolution spatial and temporal analysis of 6PPD-Q hotspots provides a tool for prioritizing the locations, amounts, and types of green infrastructure that can most effectively reduce 6PPD-Q stream concentrations to levels protective of coho salmon and other aquatic species.

## 1 Introduction

Coho salmon (*Oncorhynchus kisutch*) are highly sensitive to the stormwater runoff contaminant 6PPD-Quinone (6PPD-Q), a transformation product of the ubiquitously used tire rubber antiozonant 6PPD (Tian et al., 2021; Tian et al., 2022a). Stormwater contaminants are widely known to degrade the ecological health of urban receiving waters (Pal et al., 2014; Kaushal et al., 2020). Urban surface runoff transports contaminants from sources such as oils, pesticides, fertilizers, and animal waste along roadways to stormwater systems, treatment facilities, surrounding soils, or directly into urban streams, estuaries, and other receiving waters. Contaminants of emerging concern (CEC) can be directly discharged into streams and may not be effectively treated by stormwater management systems or wastewater treatment plants (WWTP), and they can thereby negatively impact aquatic organisms in receiving waters (Challis et al., 2021; Peter et al., 2022a). CECs are contaminants that are often not effectively treated because though they are “increasingly being detected at low levels in surface water” there is “concern that these compounds may have an impact on aquatic life”, yet “may require testing methodologies not typically available along with endpoints not previously evaluated using current guidelines” (US EPA, 2024).

One such CEC is 6PPD-Q, a transformation product of the tire rubber antioxidant 6PPD, (N^1^-(4-Methylpentan-2-yl)-N^4^-phenylbenzene-1,4-diamine)), which has been recently identified as the primary cause of a long-observed acute urban runoff mortality syndrome (URMS) in coho salmon within urban streams draining to the Puget Sound National Estuary (Tian et al., 2021; Environmental Protection Agency EPA, 2022a; Environmental Protection Agency EPA, 2022b). Existing knowledge of the toxicological and environmental fate and transport profile of 6PPD-Q remains limited. While 6PPD-Q acute toxicity to coho salmon has been estimated, toxicity to other salmonids, other fish, and aquatic biota remains under investigation (Tian et al., 2021; Tian et al., 2022a; Brinkmann et al., 2022). A 6PPD-Q review by Bohara et al. is revealing to how ubiquitous this CEC is within nature and points out that the CEC is not only toxic to coho salmon but also other aquatic species (Bohara et al., 2023).

Ongoing 6PPD-Q research has refined the knowledge base regarding the environmental factors controlling spatial and temporal tire road wear particle (TRWP) deposition within urban watersheds to receiving waters (Unice et al., 2013; Johannessen et al., 2022; Zeng et al., 2023). Urban stormwater systems are a source of 6PPD-Q and many other CECs (Unice et al., 2013; Tian et al., 2021; Tian et al., 2022a; Zeng et al., 2023). Coupling this evidence with green stormwater infrastructure (GSI) as an effective remediation tool is essential for identifying science-based stormwater management practices capable of reducing contaminant loads in receiving waters (Lundin et al., 2019).

To address this need, we applied the US Environmental Protection Agency (EPA) Visualizing Ecosystem Land Management Assessments (VELMA) ecohydrology model to characterize spatial and temporal patterns of daily and interannual TRWP deposition and associated fate and transport of 6PPD-Q in a highly urban watershed. Longfellow Creek upper watershed (5.54 km^2^), Seattle, Washington (WA), United States (US) is a well-monitored Puget Sound tributary with high rates of documented coho salmon URMS (Scholz et al., 2011; McKane et al., 2014a; Feist et al., 2017; McKane et al., 2020). Since the discovery of 6PPD-Q as a toxic stormwater pollutant, the Puget Sound region has enhanced focus on stormwater road runoff through investments in contaminant monitoring, containment, and remediation (Puget Sound Partnership, 2023).

Prior VELMA research addressed the model’s ability to simulate urban watersheds; specifically, the characterization of the Longfellow Creek urban watershed (Halama et al., 2023). That watershed setup was leveraged here due to the simulation producing high quality daily hydrologic results when compared against the King County, WA observed streamflow data (Halama et al., 2023). Here we present expanded research demonstrating fate and transport results that elucidate ecohydrological controls on 6PPD-Q flow paths, as well as 6PPD-Q contaminant hotspots within the watershed and its stormwater infrastructure, representing both Municipal Separate Storm Sewer System (MS4) and Combined Sewer System (CSS), that transport contaminants to Longfellow Creek and subsequently to Puget Sound.

Details of the hydrological and biogeochemical processes controlling 6PPD-Q fate and transport from points of deposition to streams and estuaries are poorly understood. VELMA’s high-resolution spatial and temporal analysis of contaminant movement will provide a basis for prioritizing the locations, amounts, and types of green infrastructure that can most effectively reduce contaminant stream load concentrations to levels protective of coho salmon and other aquatic species. This research focuses on applications of the VELMA ecohydrological model for assessing the model’s capability of estimating the fate and transport of 6PPD-Q. The longer-term aspiration is providing stormwater managers an ecosystem services tool capable of informing on the effectiveness of GSI improvements for reducing urban stormwater contaminant loads impacting salmonids, orca, and other biota in freshwater and estuarine habitats in the Puget Sound basin.

## 2 Methods

### 2.1 Site description

**FIGURE 1:**
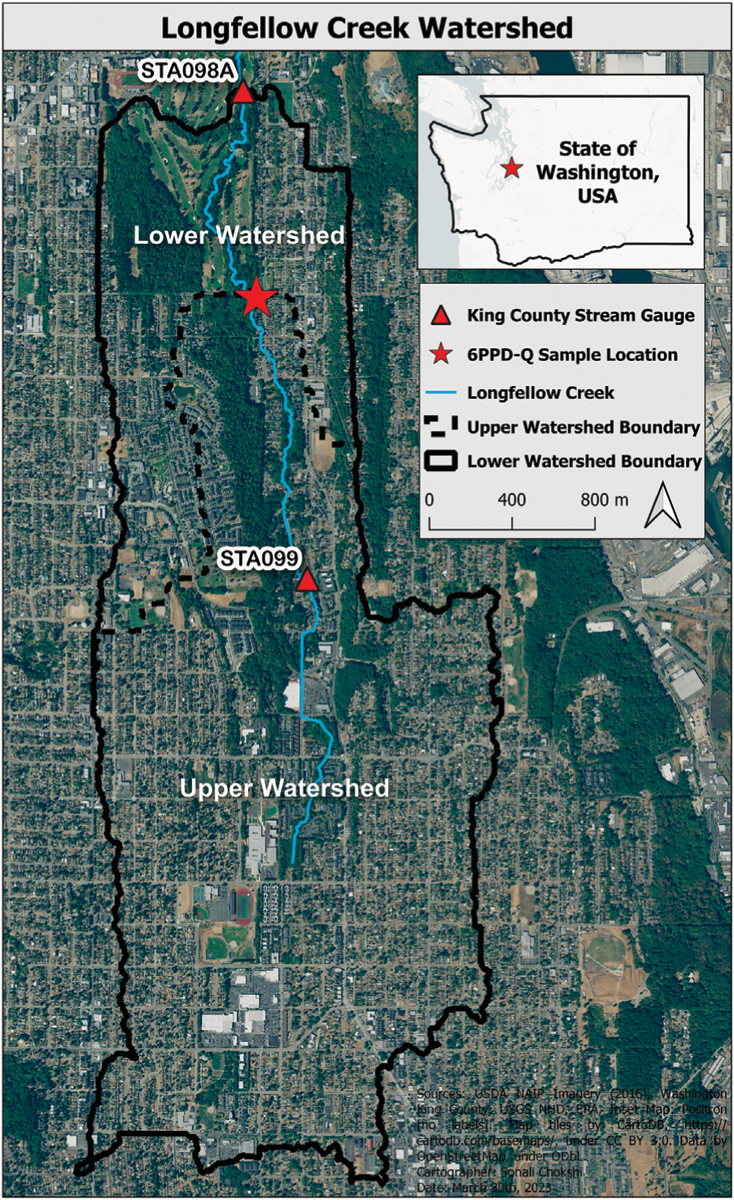
Longfellow Creek and watershed boundary in West Seattle, WA. Red star indicates Longfellow Creek 6PPD-Quinone water sample location (latitude 47.55361, longitude −122.36611). The upper watershed study area above King County STA099 stream gauge is 5.54 km^2^.

The Longfellow Creek watershed is a major portion of Seattle Council District 1 that includes West Seattle and South Park Seattle, WA, US (Figure 1). We selected this watershed due to 1) high rates of coho salmon URMS, and 2) well documented key infrastructure and monitoring data (Soundkeeper, 2017; King County, 2022). Coho salmon URMS has been intensively monitored in Longfellow Creek since 2002 (Halama et al., 2023). For example, pre-spawn mortality of female coho (an established metric of URMS occurrence in urban receiving waters) was 60%–100% of each fall run during 2002–2009 (Soundkeeper, 2017). The City of Seattle maintains publicly accessible geographic information system (GIS) data describing stormwater infrastructure (MS4 and CSS), including storm drain, lateral pipe, mainline pipe, and outflow locations (Seattle Public Utility SPU, 2022a; Seattle Public Utility SPU, 2022b). Individual segments of stormwater pipe inlets and outlets are labeled through a numbering arrangement that enabled spatially accurate mapping of stormwater infrastructure features within the VELMA model (Seattle Public Utility SPU, 2022a; Seattle Public Utility SPU, 2022b; Halama et al., 2023). Also, since 2014, King County has maintained two Longfellow Creek stream gauges in the upper and lower watershed to monitor daily streamflow (King County, 2022). The multi-year streamflow data for the STA099 gauge station facilitated VELMA hydrologic performance tests used in a companion study that underpins the 6PPD-Q fate and transport modeling work reported here (Halama et al., 2023).

The pour point (lowest location water flows out of the simulated delineation boundary) for the Longfellow Creek upper watershed simulation is the same location water samples were collected in the field and subsequently analyzed to determine 6PPD-Q concentration. Therefore, the study area for the 6PPD-Q fate and transport modeling work reported here is restricted to this 5.54 km^2^ upper Longfellow Creek watershed (Figure 1). Within the Longfellow Creek simulation delineation there are 46 Seattle Public Utility (SPU) stormwater system outfall locations upstream of the King County STA098a gauge station pour point. We mention this under the site description to highlight only the outfall locations upstream of the sampling location effect 6PPD-Q results (Figure 1). Among the 46 outfall locations, 33 outfall locations are upstream of the 6PPD-Q grab sample location (King County, 2022). The other 13 outfalls are between the King County STA098a gauge station and 6PPD-Q grab sample location, therefore do not contribute to either the observed or modeled 6PPD-Q values (Seattle Public Utility SPU, 2022a).

### 2.2 Observed 6PPD-Q stream concentrations

Water samples analyzed to produce the observed 6PPD-Q stream concentration data at the Longfellow pour point (47.55361, −122.36611), located where SW Brandon Street crosses above Longfellow Creek, are listed as follows (Figure 1; Table 1).

Stream sample collection methods and analysis of 6PPD-Q by liquid chromatography coupled to high-resolution mass spectrometry (LC-HRMS) followed those described by Tian et al. (Tian et al., 2021; Tian et al., 2022a). Grab samples (4 L) were collected during the rising hydrograph of the storm. Samples (200 mL) were extracted by solid phase extraction (200 mg, 6 mL Oasis HLB; Waters, MA), then eluted with methanol (4 × 2.5 mL). Eluates were concentrated to 1 mL under N_2_ and transferred to autosampler vials. An isotopically labeled standard for 6PPD-Q to estimate isotopic dilution and sample recovery was not available at the time of sample extraction. Thus, samples were post-spiked (in the autosampler vial) with 6PPD-Q-d5 and re-analyzed to estimate concentrations. However, reported concentrations remain semi-quantitative, and likely under-estimate true concentration given previously reported ~60–70% recovery of 6PPD-Q (Table 1) (Tian et al., 2021; Tian et al., 2022a). In 2022 Tian et al. reported in a follow-on publication “*when applied to analysis of baseflow and stormwater samples in receiving water, method limits of quantification were 2.5 and 5.1 ng/L, respectively*”. In this reporting, Tian recommended “*robust methods will be needed to maintain performance even at concentrations of* ≤ *10 ng/L”*, especially in light of the complex and dynamic stormwater matrix typical of these samples (Tian et al., 2022b).

### 2.3 Model description

Contaminant fate and transport in urban watersheds is notoriously difficult to model due to complex patterns of land use, impervious surfaces, and stormwater management infrastructure that collectively alter natural hydrologic and biogeochemical processes (Mikelonis et al., 2018). To model these complexities, we enhanced the VELMA model to explicitly simulate urban landscape features at high spatial resolutions and their impact on watershed hydrology (Halama et al., 2023). VELMA is a spatially explicit (grid-based) ecohydrology model that dynamically integrates watershed hydrological and biogeochemical processes across multiple scales. VELMA includes hydrologic and biogeochemical controls on contaminant fate and transport and is applicable to essentially any land cover type (e.g., urban, agricultural, forest, grassland, prairie, wetland, alpine) (Abdelnour et al., 2013; McKane et al., 2014b). Recent innovative research has advanced VELMA methodology and techniques to simulate mixed-use watersheds characteristic of urban and suburban environments (Hoghooghi et al., 2018; Barnhart et al., 2021; McKane R. B. et al., 2022; Oregon Department of Fish and Wildlife ODFW, 2022). Prior to these improvements, the VELMA models’ fate and transport capabilities were limited to nonurban settings for mercury and the nutrients ammonium, nitrate, dissolved organic nitrogen, dissolved organic carbon (Golden et al., 2012; Abdelnour et al., 2013; Golden et al., 2013; Golden et al., 2014; Knightes et al., 2014).

We enhanced VELMA in two essential ways to model contaminant fate and transport within urban watersheds. First, the Piwoni and Keeley (1990) method was incorporated into VELMA for simulating hydrological and biogeochemical processes by which contaminants are partitioned between sorbed (bound by soil) and aqueous (dissolved) phases on impervious surfaces or within soil columns (Piwoni and Keeley, 1990; McKane et al., 2014b). To apply this approach, users assign contaminant-specific environmental fate and transport parameters described for any of hundreds of thousands of contaminants listed in the EPA CompTox library (Williams et al., 2017; Environmental Protection Agency EPA, 2022a). CompTox provides curated values for contaminant solubility, hydrophobicity, partitioning between aqueous and sorbed phases, decay rate, and other properties. These parameters allow dynamic daily simulation of changing hydrological and biogeochemical controls when utilized within a calibrated watershed (McKane et al., 2020; McKane R. B. et al., 2022). Depending on variability in soil matrixes, contaminants can accumulate through cell-to-cell transfers at surface and subsurface flow path convergences and then be transported to stormwater outfalls along streams, estuaries, or other receiving waters. VELMA supports spatial and temporal visualizations of these surface and subsurface contaminant fate and transport dynamics.

The second important VELMA enhancement was the inclusion of spatially explicit physical features of urban watersheds required to realistically simulate deposition and runoff of urban stormwater pollutants. These included 1) stormwater gray infrastructure like spatially explicit drains, pipes, and outlets for MS4 and CSS, and 2) impervious surfaces such as roads with or without curbs, roofs with or without lateral drains to MS4 or CSS systems, and parking lots. Longfellow Creek watershed simulation setup details that included these urban features and resulting simulated stream outflows are presented in Halama et al. (Halama et al., 2023). Spatial representation of the gray infrastructure stormwater inlets was crucial for accurately representing water transport along roadways and through the stormwater system to outfalls. Explicit MS4 outfall representation enables accurate contaminant fate and transport simulation for roadway deposited contaminants.

Given the fine spatial scale (5-m) required to understand these transfers and associated chemical transformations in complex urban watersheds, VELMA has also been enhanced to track daily transfer of simulated contaminant(s), then record those daily results as a spatial ASCII grid file. To visualize these detailed results data, VISTAS software was implemented to produce spatially distributed, daily VELMA visualizations (Cushing et al., 2015).

### 2.4 Setup 6PPD-Q deposition for fate and transport

**FIGURE 2:**
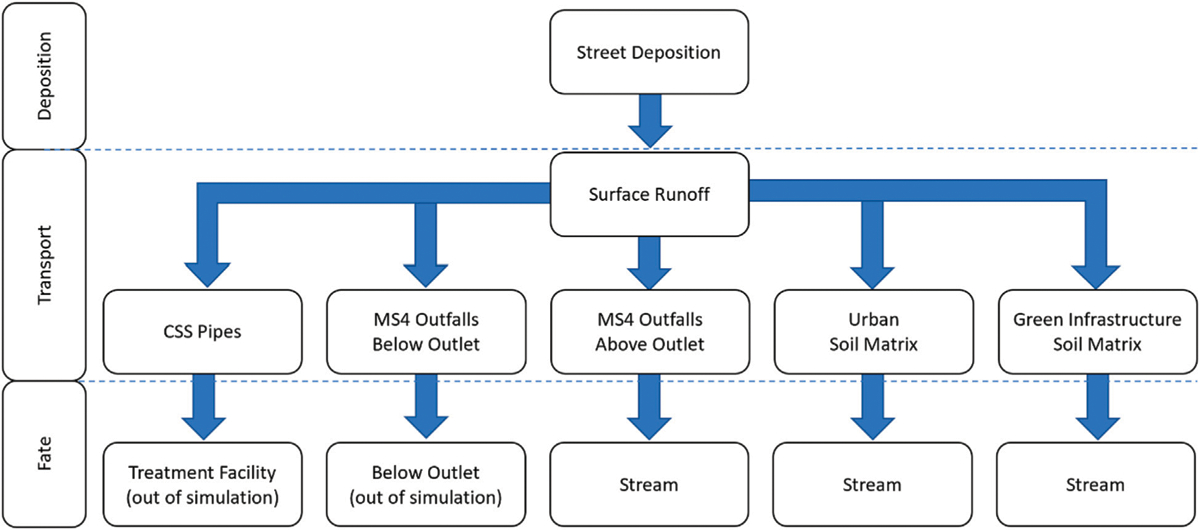
Conceptualization of the integration of spatial data in VELMA for modeling 6PPD-Q fate and transport in the Longfellow Creek watershed. Arrows indicate potential 6PPD-Q transfers into, out of, and within grid cells and whole watershed.

The following methods were used to set up VELMA to model 6PPD-Q fate and transport in the Longfellow Creek watershed. Integration of these enhancements in VELMA provided the capability to model watershed 6PPD-Q fate and transport across wide spatial and temporal scales. VELMA modeling components contribute to simulating 6PPD-Q transport and associated net gains and losses at the scales of individual 5-m grid cells to whole watershed (Figure 2). VELMA tracks these spatial gains and losses daily to calculate and map daily and annual 6PPD-Q mass balances.

The simulation of a contaminant within VELMA is partitioned into several components. Methods regarding contaminant deposition, transport, and parameterization are detailed in subsections 2.4.1 through 2.4.3 (Figure 2).

1. VELMA urban spatial data (GIS) setup and model calibration (Halama et al., 2023).
2. Model Performance Tests: Hydrology daily runoff and annual results (Halama et al., 2023).
3. Simulating daily deposition of 6PPD-Q on roadway cells.
4. VELMA parameterization for simulating 6PPD-Q fate and transport.

Steps 1 and 2 strictly involve the hydrology setup and have been described previously, as cited, but are listed here for completeness. Step 3 details how 6PPD-Q was deposited within the model framework, and step 4 specifies the properties of the 6PPD-Q contaminant within the model.

#### 2.4.1 Urban spatial data (GIS) setup and model calibration

The Longfellow Creek watershed sources and methods used to set up the urban spatial data layers and weather driver data are fully described by Halama et al. (Halama et al., 2023). That work established the hydrologic basis for modeling 6PPD-Q fate and transport within the Longfellow Creek watershed. Assessment of roads, buildings, stormwater systems, and GSI in GIS revealed a 5-m grid is a sufficient scale for modeling urban hydrology and chemical fate and transport based on the minimal width of listed landscape features. Halama et al. summarized the major urban spatial components developed for representing urban terrain features to achieve suitable hydrography within VELMA’s framework (Halama et al., 2023).

#### 2.4.2 Simulating daily deposition of 6PPD-Q on roadway cells

Fitting to VELMA’s existing disturbance framework, an approach was developed for depositing daily 6PPD-Q onto watershed roadways. Within the model framework 6PPD-Q is deposited upon the roadway even though in reality 6PPD (not 6PPD-Q) is deposited on roadways and breaks down to 6PPD-Q. We use the term “deposition” henceforth to describe our approach for adding the contaminant 6PPD that degrades to 6PPD-Q by accounting for the chemical degradation at deposition, *versus* internally within the model, at rates consistent with current knowledge (Kole et al., 2017). The resulting deposition estimates can easily be adjusted as new empirical evidence emerges (Table 2).

The watershed maximum possible daily deposition is calculated through conversion from TRWP deposition (mg km^−1^ day^−1^) to 6PPD-Q deposition (g m^−2^ d^−1^). A unit conversion from TRWP (mg km^−1^ day^−1^) to TRWP_ADJ_ (g m^−1^ day^−1^) is performed to convert units to grams (Eq. (1); Table 2).


(1) $${\text{TRWP}}_{\text{ADJ }}\left({\text{g m}}^{-1}{\text{ day}}^{-1}\right)=\frac{\text{TRWP}\left(\text{mg }km^{-1}day^{-1}\right)\times 1000\left(\frac{m}{km}\right)}{1000\;\frac{g}{mg}}$$

TRWP_ADJ_ is converted to TRWP_PIXEL_ (g m^−2^ day^−1^) to account for the estimated TRWP linear cell resolution distance of tire wear deposition across a 2D cell area within the model framework (Eq. (2); Table 2).


(2) $${\text{TRWP}}_{\text{PIXEL }}\left({\text{g m}}^{-2}{\text{ day}}^{-1}\right)=\frac{TRWP_{ADJ}\times \text{ cellResolution }(\text{m})}{\text{ cellArea }\left(m^{2}\right)}$$

The maximum possible quantity of 6PPD-Q contaminant deposition is calculated from the TRWP_PIXEL_ value where TC_FRACTION_ is the percentage of the tire material that is 6PPD, CC_FRACTION_ is the percentage of 6PPD that transforms to 6PPD-Q, and α is a calibration parameter that is available but not used in this study (i.e., used here at the default value of 1.0) (Eq. (3); Table 2).


(3) $${\text{TRWP}}_{6\text{PPDQ}}\left({\text{g m}}^{-2}{\text{ day}}^{-1}\right)=TRWP_{\text{PIXEL}}*TC_{\text{FRACTION}}*CC_{\text{FRACTION}}*\alpha$$

**FIGURE 3:**
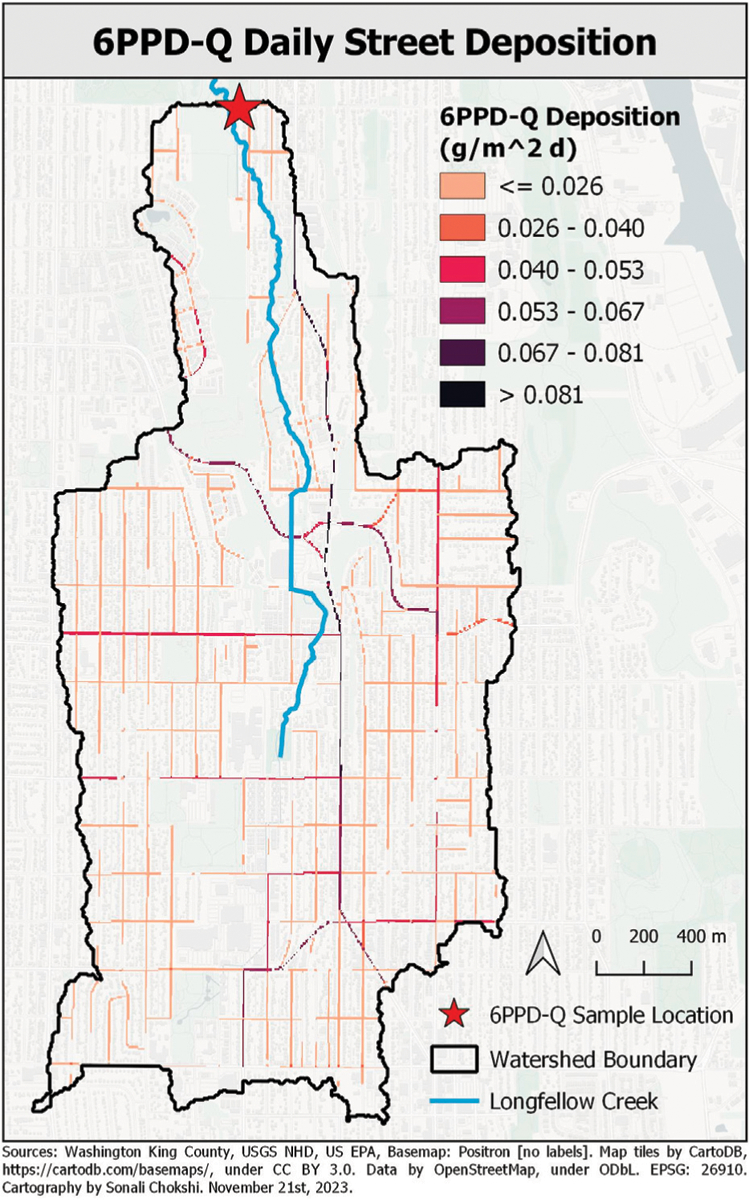
Spatial pattern of 6PPD-Q deposition (g m-2 days-1) on roads within watershed, based on method presented in S4 panels A, B, C and described in S5.

Research presented here used the values listed in Table 2 to calculate the maximum possible quantity of 6PPD-Q contaminant deposition (Table 2). With the watershed specific TRWP_6PPDQ_ determined, the distribution of contaminant was calculated for all other roadway cells. The following describes how traffic pattern count data, the roadway network within the watershed, and the TRWP_6PPDQ_ were used to determine a contaminant deposition distribution. Here the final 6PPD-Q contaminant deposition data are presented (Figure 3). Each step of the workflow are presented in the Supplementary Material S4A–C. The automated Python code for executing the task is also provided in the Supplementary Material S5.

The publicly available SPU Streets GIS dataset included all roads within the Longfellow Creek watershed (S4-A) (Seattle, 2022a). The 2018 Traffic Flow dataset represented a subset of monitored roads providing an estimate for traffic counts (S4-A) (Seattle, 2022b). For all road segments not represented with a traffic count value (typically neighborhood streets and side roads), traffic count values were assigned based on a GIS comparison to similar neighborhood streets and side roads streets that did contain a traffic count value (S4-B). Python code used for generating a VELMA 6PPD-Q deposition CSV file was provided as Supplementary Material S5.

The spatial data representing an estimate of traffic counts for all roadways were scaled 0 to 1 from the minimal and maximum traffic counts where 0 represents the smallest traffic count and 1 represents the highest traffic count (West Seattle Bridge in this study) (Eq. 4, S4-C).


(4) $${\text{Traffic}}_{\text{SCALAR}}=\prod_{i=0}^{n}\frac{{\text{Traffic}}_{\text{COUNT}}-{\text{Traffic}}_{\text{MIN}}}{{\text{Traffic}}_{\text{MAX}}-{\text{Traffic}}_{\text{MIN}}}$$

Daily 6PPD-Q deposition (Deposition6PPDQ (g m^−2^ day^−1^)) was calculated per cell by multiplying each cell TRWP6PPDQ value by the corresponding cells TrafficSCALAR value (Eq. (5); Table 2).


(5) $${\text{Deposition}}_{6\text{PPDQ}}\left({\text{g m}}^{-2}{\text{ day }}^{-1}\right)={\text{TRWP}}_{6\text{PPDQ}}*{\text{Traffic}}_{\text{SCALAR}}$$

The result was a spatially distributed 6PPD-Q contaminant deposition pattern across all street cells (Figure 3). Within the VELMA contaminant disturbance routine, this distribution of 6PPD-Q contaminant is deposited on the surface layer daily.

#### 2.4.3 VELMA parameterization for simulating 6PPD-Q fate and transport

Piwoni and Keeley (1990) have described parameters and equations needed to quantify process-level controls on the partitioning of organic contaminants into sorbed and aqueous phases, including the effect of soil carbon on sorption and other factors (Piwoni and Keeley, 1990). Though Piwoni and Keeley (1990) developed their approach for groundwater (aquifer) remediation applications typically characterized by low-carbon and low-permeability substrates, we adapted their procedures for application to carbon-rich, ecohydrologically active near-surface (vadose zone) soils by integrating them into VELMA (McKane R. B. et al., 2022). We made no modifications to Piwoni and Keeley (1990) equations describing contaminant partitioning between soil aqueous and sorbed phases (Piwoni and Keeley, 1990). Their work integrated within VELMA’s hydrologic and biogeochemical submodels enabled the simulation of sorption-desorption dynamics within near-surface (vadose zone) soils, such as those represented in VELMA (McKane R. B. et al., 2022).

VELMA simulates soil decomposition dynamics from CompTox-based maximum contaminant decay parameter values, adjusted for sub-optimal temperature, moisture, and soil carbon conditions. That is, under sub-optimal conditions, contaminant decay can be much longer than the specified maximum decay rate. For 6PPD (and by proxy, 6PPD-Q), the CompTox-specified maximum decay rate corresponds to a very short half-life of 3.14 days (Table 3).

Contaminant submodel parameters and values used to simulate 6PPD-Q fate and transport were obtained from the EPA CompTox database, plus select cited research (Table 3) (Hu et al., 2022). These parameters control 6PPD-Q fate and transport on VELMA’s surface layer and within four soil layers: contaminant sorption as a function of soil carbon content (*Koc*); contaminant solubility (*setMolarSolubilityCoefficient*); affinity for water as a measure of chemical hydrophobicity (*logKow*); and maximum contaminant decay rate (*setChemMaxDecay*) when soil carbon, moisture, and temperature conditions are optimal (Table 3). VELMA is designed to model sub-optimal rates of change for all these processes. Importantly, we note that several parameter values are for 6PPD, rather than 6PPD-Q, because curated values for the latter were not available at the time of final simulation execution. Given the time and research investment required to develop a panel of vetted values, the 6PPD data provides the most reasonable currently available proxy for the modeling objectives reported here. However, we acknowledge there are important 6PPD-Q *versus* 6PPD differences. For example, here the *setChemMaxDecay* parameter was calculated using the contaminants expected half-life of 3.14 days as reported per CompTox for 6PPD. 6PPD-Q half-life has yet to be reported. In VELMA the *setChemMaxDecay* value is based on ideal water moisture and temperature conditions that allows for optimal chemical breakdown rate. The simulated rate can be expected to be below the optimal breakdown rate because the environmental conditions in the Pacific Northwest region are rarely both optimally wet and warm at the same time.

## 3 Results and discussion

### 3.1 Predicted vs. observed 6PPD-Q stream concentrations

VELMA’s simulated contaminant fate and transport of 6PPD-Q in the Longfellow Creek watershed was compared against observed 6PPD-Q Longfellow Creek stream concentrations as a measure of model performance. VELMA Longfellow Creek 6PPD-Q stream concentration (ng/L) performance tests leveraged: 1) the VELMA hydrologic setup and results described by Halama et al. (Halama et al., 2023), 2) the VELMA setup for simulating 6PPD-Q deposition and fate and transport described above, and 3) the Tian et al. 6PPD-Q grab sample data (Tian et al., 2021; Tian et al., 2022a). VELMA simulated daily 6PPD-Q runoff for years 2020 and 2021 to the modeled pour point (i.e., delineation outlet) location (Figure 3). Modeled daily 6PPD-Q concentrations and observed grab sample 6PPD-Q concentrations at the pour point differed at most by 10 ng/L across the observed range of 2–27 ng/L between October 2020 and May 2021. Given that modeled concentrations are daily averages, while observed concentrations represent snapshots of concentration at a single point in time during storm events, both the observed level of agreement and modeled to observed difference falling below recommended ≤10 ng/L method limits of quantification is encouraging. These results were obtained from VELMA’s first 6PPD-Q fate and transport simulation, from roadway cells of deposition to the pour point of a highly complex 5.54 km^2^ urban watershed. Beyond the hydrologic calibration presented in Halama et al., no calibration adjustments were required here to model 6PPD-Q parameters representing deposition of TRWP, 6PPD, and transformation product 6PPD-Q, or any other modeling features (Table 3; Figure 3) (Halama et al., 2023).

**FIGURE 4:**
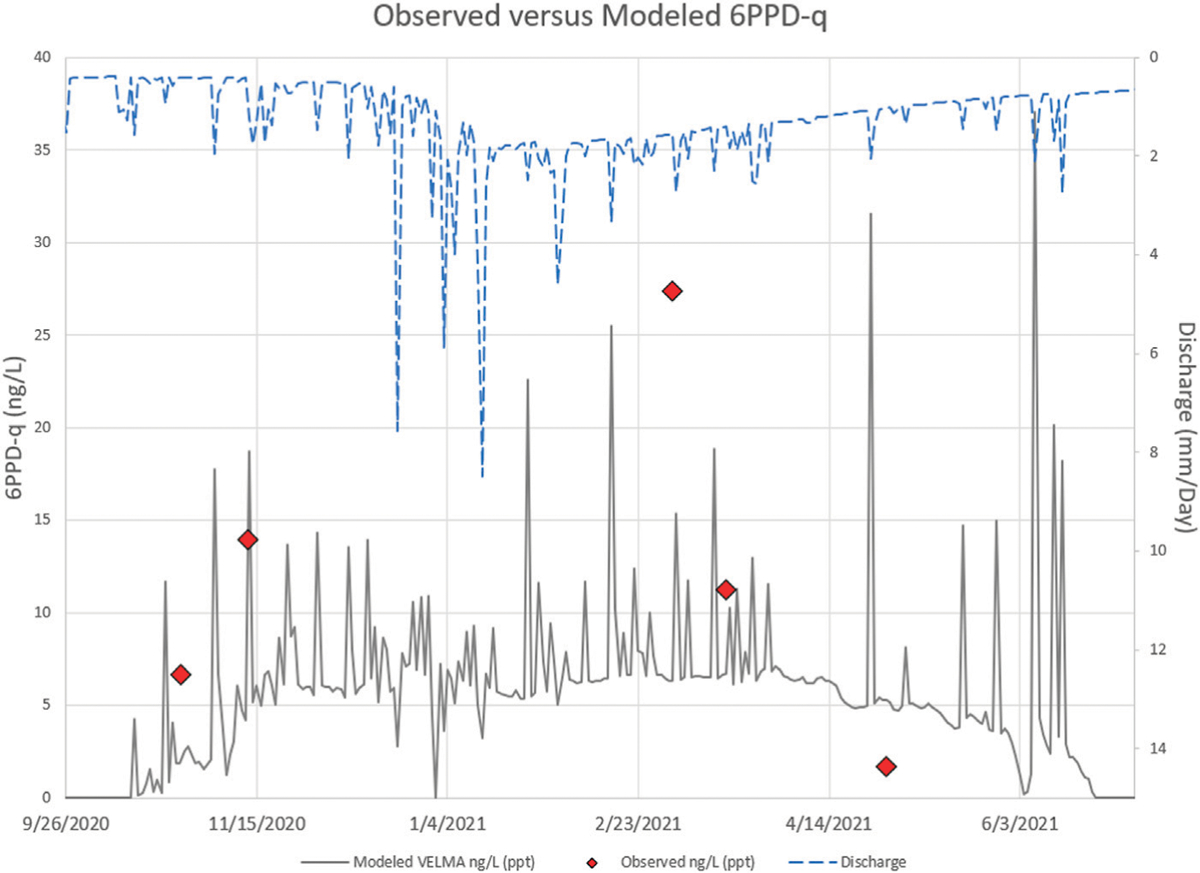
Observed grab samples *versus* simulated daily VELMA 6PPD-Q ng/L during 2020 and 2021 at the Longfellow Creek upper watershed pour point location in Figures 1, 3, plus observed 6PPD-Q values listed in Table 1.

Comparison of simulated patterns of daily 6PPD-Q concentrations and streamflow show that many of the highest simulated concentrations of 6PPD-Q occur after dry periods during which TRWP has built up on road surfaces. Then, even small rain events can mobilize contaminant mass, yielding high in-stream contaminant concentrations (Peter et al., 2022b). Lower modeled 6PPD-Q concentrations during peak storm runoff may reflect dilution and/or depletion of available 6PPD-Q on road surfaces. 6PPD-Q stream concentrations during summer months were often zero or near zero (undetectable), reflecting a near-absence of dry season surface flow as well as subsurface (baseflow) contributions to streamflow (Figure 4) (Halama et al., 2023).

All simulated and observed concentrations for the upper watershed were below the established lethal concentration 50 (LC_50_) of 95 ng/L for coho salmon (Spromberg and Scholz, 2011; Tian et al., 2021; Tian et al., 2022a). However, compared to other portions of Longfellow Creek, the traffic count data are generally lower within the Longfellow Creek upper watershed. Streets within the Longfellow Creek delineation mainly serve side streets and connecting local roadway arteries. Traffic count data in the Longfellow Creek lower (northern) watershed (S4-A) are generally much higher, especially where the West Seattle Bridge, a multilane highway serving commercial truck and commuter traffic, crosses into West Seattle from Seattle high-volume interstate, state, and county commuter and business district corridors. In addition, thirteen MS4 outfalls that transport contaminants through road runoff outflow near or into the creek below the grab sample data collection location. No observed 6PPD-Q stream data are presently available for stream reaches downstream of the grab sample location presented here (Figure 1).

### 3.2 6PPD-Q contaminant transport and distribution

**FIGURE 5:**
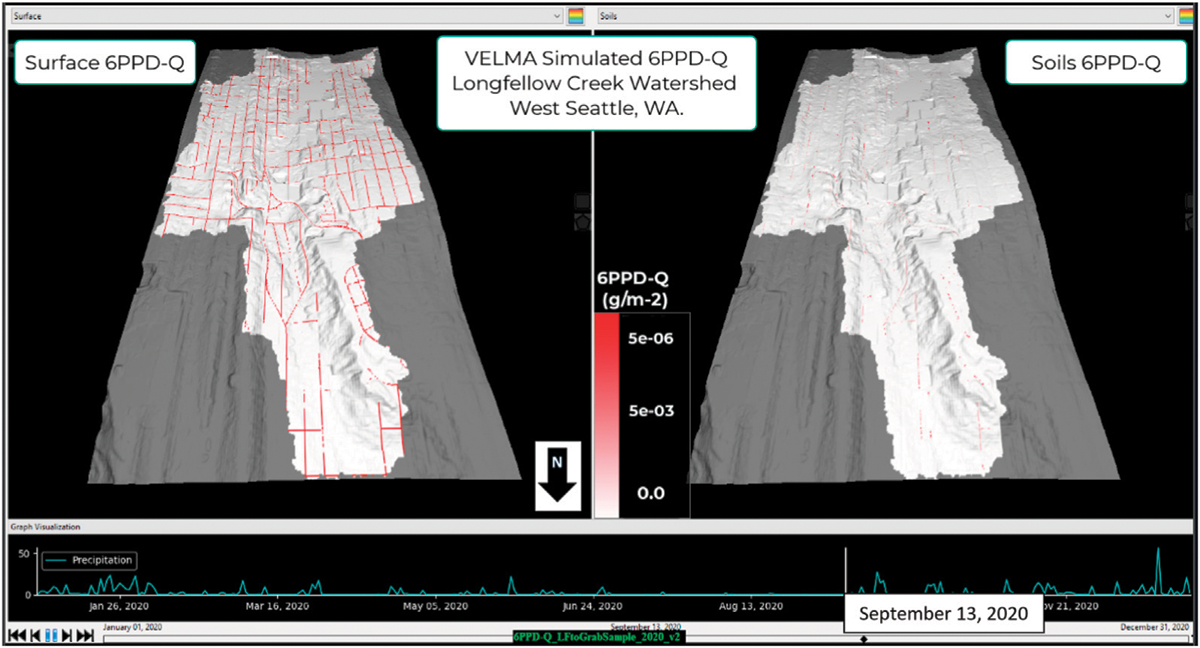
13 September 2020, VELMA-VISTAS still-frame visualization of daily 6PPD-Q (g/m2) on roads and other impervious surfaces (left panel) and in soil (right panel). Contaminant in loadings in soils are low due to the minimal summer rainfall transferring street surface buildup of contaminant into soil. See video in Supplementary Material to watch animated daily dynamics for both panels from January 1 to 31 December 2020. Daily rainfall (mm/d) during 2020 is shown graphically at the bottom of scene.

The similarity between modeled and observed 6PPD-Q stream concentrations prompt questions about the processes and flow paths controlling the amounts and timing of 6PPD-Q delivery to Longfellow Creek at the upper watershed pour point (Figure 1; Figure 4). A static frame of VELMA-VISTAS visualizations of modeled 6PPD-Q (g/m^2^) were presented here for 13 September 2020 (Figure 5, S1 Video). The left frame shows 6PPD-Q on road surfaces while the right frame shows 6PPD-Q in soils, primarily sorbed by soil carbon in the top layer of VELMA’s 4-layer soil column (Figure 5, S1 Video). 6PPD-Q concentration spatiotemporal differences are revealing when reviewing the video provided as Supplementary Material S1. When reviewing the video, we suggest focusing on 13 September 2020, *versus* 2 February 2020. The figure and Supplementary Video show the contrasting effects of seasonal differences in rainfall patterns on 6PPD-Q fate and transport within the Longfellow Creek upper watershed.

During the Pacific Northwest wet season (October–May) regular intervals of rainfall wash 6PPD-Q off roads to both streams and soils, dependent on MS4 stormwater inlet and outlet locations. Frequent winter storm events leading up to February have washed most 6PPD-Q off roads to soils at various destinations, primarily to MS4 stormwater system outfalls draining directly to grid cells near or at Longfellow Creek and to ditches catching runoff from roads lacking curbs (Figure 5, S1 Video).

High levels of 6PPD-Q transport into the soils are the consequence of the wet season (S1 Video). In contrast, during the summer dry season (June–September), 6PPD-Q deposition builds up on roads, while 6PPD-Q previously flushed to soils during the wet season continues to decompose throughout the summer, having been sorbed/immobilized in place wherever there are sufficient stocks of soil carbon (Figures 5 right panel). Simulated results reveal the buildup of 6PPD-Q on road surfaces near the end of the summer dry season (September 13th), and correspondingly low amounts of 6PPD-Q in soils (Figure 5). Thus, this annual wet/dry season cycle leads to alternating pulses in the levels of 6PPD-Q on roads and in soils.

Animated daily surface transfers include visual disappearance into storm sewer (MS4 and CSS) drains and pipes, and re-emergence at outfall locations, often onto riparian soils where the dynamic daily and seasonal balance of sorption-decomposition dynamics are apparent (S1 Video). The parameter *setChemMaxDecay* (set based on known behavior of 6PPD) yields a high maximum decay rate under optimal conditions in soil for modeled 6PPD-Q (Table 3). VELMA’s underlying fate and transport design demonstrates dynamic movement of 6PPD-Q contributing to the consequent net daily changes in soil 6PPD-Q captured in the video animation (S1 Video).

## 4 Modeling uncertainty

Work presented here is a proof-of-concept demonstration that a spatially distributed, process-based ecohydrology model can predict the fate and transport of a highly toxic contaminant, 6PPD-Q. As a recently discovered contaminant, the 6PPD-Q work reported here was largely accomplished using parameters for its proxy 6PPD, providing values having an unknown degree of alignment (Tian et al., 2021; Tian et al., 2022a). As 6PPD-Q is further studied, the extent of these uncertainties will likely narrow. For now, while the spatial and temporal patterns of modeled 6PPD-Q fate and transport appear realistic, further laboratory and field research are needed to estimate spatial and temporal uncertainties near and far from the sole 6PPD-Q stream sample location. The current potential sources of uncertainty in our VELMA simulations, and our strategies for constraining those, are summarized here:

- Limited observed data: within Longfellow watershed the number of 6PPD-Q stream sample data available to date for model performance tests are limited to five single day samples between October 2020 and April 2021. This should be considered a bare minimum, while recognizing that 6PPD-Q had only been discovered immediately before the 2020 stream sampling dates (Mikelonis et al., 2018; Tian et al., 2022a). Hydrologic parameters: modeled 6PPD-Q fate and transport within the watershed can be no more accurate than the quantities and routing of water within the simulated engineered and natural infrastructure controlling those hydrologic dynamics. For this study, VELMA hydrologic parameter values (presented in Halama et al.) were calibrated against stream gauge data prior to 6PPD-Q simulations, such that hydrologic parameters were not used to calibrate 6PPD-Q stream concentrations (Halama et al., 2023).
- Contaminant environmental fate and transport values: here we relied upon current knowledge of 6PPD-Q chemistry. 6PPD mid-range values were used as a proxy when a 6PPD-Q value was unknown. No contaminant parameter value was calibrated to improve model results.
- 6PPD Tire Mass: the percent of tire mass that is 6PPD was simulated as 2%. Tian et al. (2021) reported values ranging from 0.4 to 2 percent for automobiles (Babbit, 2010). Li et al., demonstrated the importance of photodegradation as a “non-negligible source of the highly toxic 6PPDQ compound in surface water” (Li et al., 2023).
- Chemical conversion: the percent of 6PPD transformed to 6PPD-Q has a reported theoretical range of 1%–75% (Tian et al., 2021; Tian et al., 2022a). Here the mid-range value of 38% was used to calculate 6PPD-Q deposition. Since 2020, authors have experimentally observed values of 20% or less, therefore a lower parameter value is likely more realistic.
- Street deposition spatial pattern: 6PPD surface deposition pattern was determined from SPU traffic count data (Seattle, 2022b). The deposition quantity per roadway pixel is scaled to match the traffic pattern, but neither seasonal driving patterns nor heavy truck to light car ratio are represented in the final deposition data. In addition, the focus of this research was modeling on street contaminant deposition migration into stormwater systems and ultimately into aquatic systems. Within VELMA’s framework only street pixels linking in a chain upslope of a street storm drain inlet were included in the contaminant street deposition pattern. Roads not meeting that upslope criteria and all parking lots were not simulated as having 6PPD-Q deposition.
- Airbourne particles: dispersion of TRWP and resulting 6PPD-Q was not included as deposition in these model simulations. Researchers are discovering airborne 6PPD-Q as a microplastic pollution of concern regarding inhalation by animals and humans spending a considerable amount of time near highways (Olubusoye et al., 2023).

The sensitivity of several contaminant deposition parameters were presented as Supplementary Material S2, S3. Multiplicative parameters exhibit a logical response to the magnitude of contaminant deposition and therefore resulting 6PPD-Q at the pourpoint, as well as the parameter controlling the half-life of 6PPD-Q.

## 5 Environmental implications

The goal of this study was to use the VELMA ecohydrology model to accurately characterize spatial and temporal patterns of daily TRWP (and corresponding 6PPD-Q) deposition, and subsequent fate and transport of remaining contaminant into the aquatic stream habitat. The highly urbanized upper Longfellow Creek watershed was used as the example hydrologic bounds to demonstrate 6PPD-Q contaminant transport from street depositions to Longfellow Creek where 6PPD-Q concentrations are known to cause high rates of acute URMS for fall runs of coho salmon.

We leveraged the spatial data, temporal data, and parameterization incorporating watershed climate drivers, flow paths, land cover, soils, impervious surfaces, and stormwater infrastructure as inputs into this VELMA ecohydrological model setup (Halama et al., 2023). We incorporated 6PPD-Q contaminant depositions via TRWP distribution within the model to simulate the fate and transport of this highly toxic CEC in an urbanized environment (Figure 3). Due to the rapid pace of CECs chemical properties research, one goal for including contaminants within VELMA was parameter simplicity and flexibility. Lacking most published model parameters (soil adsorption coefficient, maximum decay rate, and solubility coefficient) for 6PPD-Q, curated model parameters for 6PPD were applied to VELMA to fill parameter knowledge gaps (Table 3).

The integration of all this information in VELMA enabled accurate simulation of daily and interannual hydrologic responses and 6PPD-Q stream concentrations in Longfellow Creek (Halama et al., 2023). Compared to observed concentration data (n = 5 days during 2020–2021), simulated stream concentration errors were within the same magnitude of the observed 6PPD-Q grab sample data (Figure 4).

VELMA’s ability to track and visualize daily transport of 6PPD-Q on roadways and in stormwater pipes draining to various destinations (i.e., creek, riparian soils, ditches, WWTP) made it possible to quantify and visualize spatial and temporal dynamics of daily and annual 6PPD-Q hotspots on roads and soils (Figure 5, S1 Video). Directing contaminants to GSI, such as bioswales, may provide suitable remediation because 1) strong sorption of 6PPD-Q to soil carbon effectively arrests further lateral and vertical transport within the watershed, and 2) decomposition of sorbed 6PPD-Q could eliminate it from the watershed.

## 6 Conclusion

To summarize, the research reported herein outlines a model-based approach for characterizing spatial and temporal patterns of daily and interannual TRWP deposition and subsequent fate and transport of the 6PPD transformation product 6PPD-Q within an urbanized watershed. VELMA’s high-resolution spatial and temporal analysis of 6PPD-Q fate and transport, and potentially other CEC’s, provides a tool for prioritizing the locations, amounts, and types of green infrastructure that can most effectively reduce stream contaminant concentrations to levels protective of coho salmon and other aquatic species.

Chief advantages of this modeling approach include its capabilities to 1) accurately model 6PPD-Q delivery and stream concentrations for an urban creek experiencing high rates of coho salmon URMS, and 2) pinpoint 6PPD-Q hotspots at a scale of 5-m and provide process-based insights pertinent to the identification of practical GSI-based remediation best practices. Such information would be extremely difficult to obtain without a process-based model (Martin-Mikle et al., 2015).

Chief disadvantages for those new to VELMA are the time and effort required to learn and implement the model for complex urban landscape applications. VELMA training materials and workshops have been initiated to facilitate this process (Pan et al., 2012; McKane et al., 2014b; McKane R. et al., 2022).

If this mechanistic VELMA-based approach can be applied across a sufficient range of watershed types within a region or regions, it may be possible to develop broadly applicable guidelines and simpler tools than VELMA for more quickly identifying likely contaminant hotspots and GSI best management practices. For example, easily applied digital elevation model (DEM) processing tools are already available to identify dominant flow paths within watersheds. JPDEM is one such tool developed and used to prepare DEM flow path maps for VELMA (Pan et al., 2012; McKane R. B. et al., 2022; McKane R. et al., 2022). When coupled with easily obtained flow path and traffic count data, VELMA results for a subset of intensively modeled watersheds could potentially be used to develop relatively simple and transferable GIS-based methods adequate for estimating dominant contaminant hotspot locations, independently of VELMA. This GIS-based approach would need to leverage GIS staff associated with most urban municipalities or their stormwater management partners.

VELMA’s high-resolution spatial and temporal analysis of 6PPD-Q hotspots demonstrated the capabilities of this tool and approach for prioritizing the locations, amounts, and kinds of GSI that can most effectively reduce 6PPD-Q stream concentrations to levels protective of coho salmon and other aquatic species. This work represents a starting point for using VELMA to inform ongoing 6PPD-Q and other CEC remediation efforts in the Puget Sound region by community, tribal, state, and federal partners.

## Supplementary Material

Supplement1

## Figures and Tables

**TABLE 1:** Observed 6PPD-Q sample dates and assessed quantity.

Sample #	Sample date	6PPD-Q (ng/L)
1	26 October 2020	6.63
2	13 November 2020	14.3
3	04 March 2021	27.4
4	18 March 2021	11.2
5	29 April 2021	1.71

**TABLE 2:** Parameters for estimating contaminant deposition.

Parameter	Units	Value
TRWP	mg km^−1^ day^−1^	100
cellResolution	meters	5
TC_FRACTION_	0 to 1 (unitless)	0.02
CC_FRACTION_	0 to 1 (unitless)	0.38
α	0 to 1 (unitless)	1.0

**TABLE 3:** VELMA 6PPD/6PPD-Q contaminant parameters and values.

Parameter name	Type	Value	Description
directKocSpecification	Model	True	Set = *True* when the value of Koc is known for a contaminant (check the EPA CompTox dashboard). When Koc is unknown, set = *False* and use alternate VELMA parameters and methods described in McKane et al. (2022a) to estimate Koc
Koc	CompTox Parameter (6PPD)	11,000 L/kg	Contaminant soil adsorption coefficient
setMolarSolubilityCoefficient	CompTox Parameter (6PPD)	0.000158 mol/L	Molar solubility coefficient (mol/L)
setMolarMass	CompTox Parameter (6PPD-Q)	268.404 g/mol	Molar mass (g/mol). Value is used to internally convert mol to g
setChemMaxDecay	CompTox Parameter (6PPD)	0.2,207,475	Maximum contaminant decay rate (fraction per day)
logKow	Hu et al., (Seattle, 2022a) Parameter (6PPD-Q)	2.745	Octanol-water partition coefficient (unitless)

## Data Availability

The original contributions presented in the study are included in the article/Supplementary Material, further inquiries can be directed to the corresponding author.
