# Supplementary figures and images for: Watershed analysis of urban stormwater contaminant 6PPD-Quinone hotspots and stream concentrations using a process-based ecohydrological model
Source: Front Environ Sci. Author manuscript; Available in PMC 2025 Mar 6. (PMC11151736; doi:10.3389/fenvs.2024.1364673)

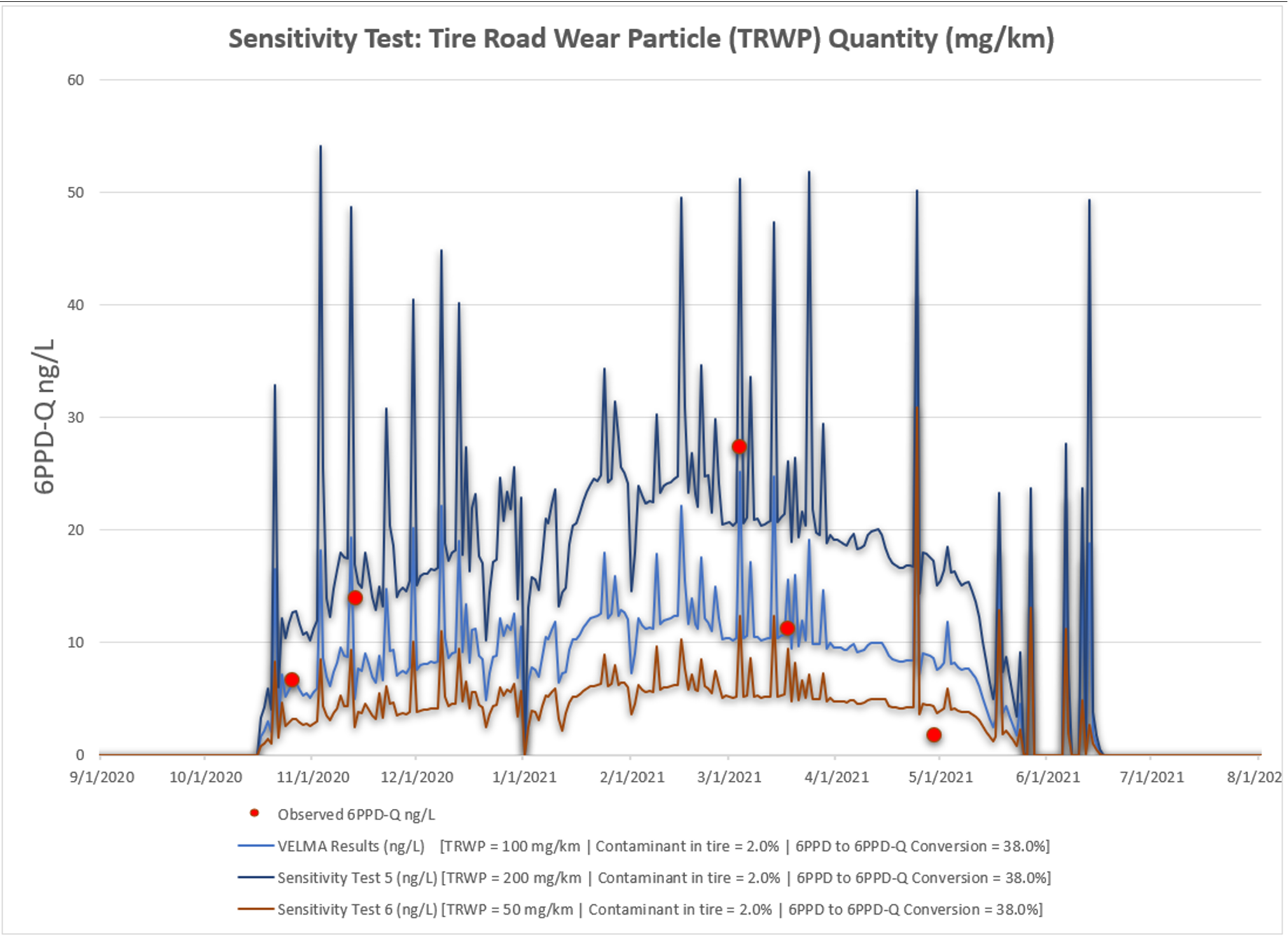

Supplement: Supplement1 [file NIHMS1974508-supplement-Supplement1.zip › Image 1.TIFF]

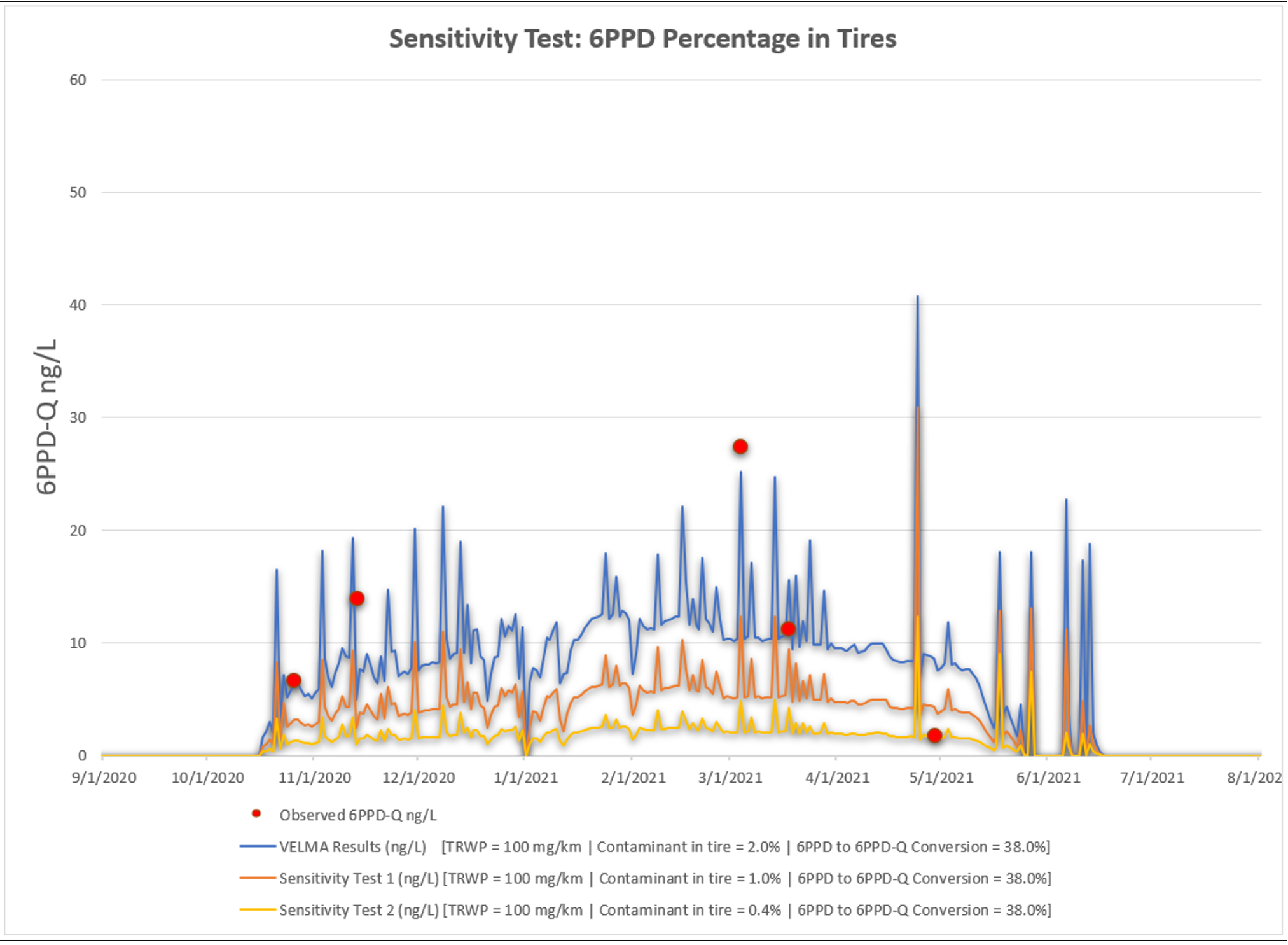

Supplement: Supplement1 [file NIHMS1974508-supplement-Supplement1.zip › Image 2.TIFF]

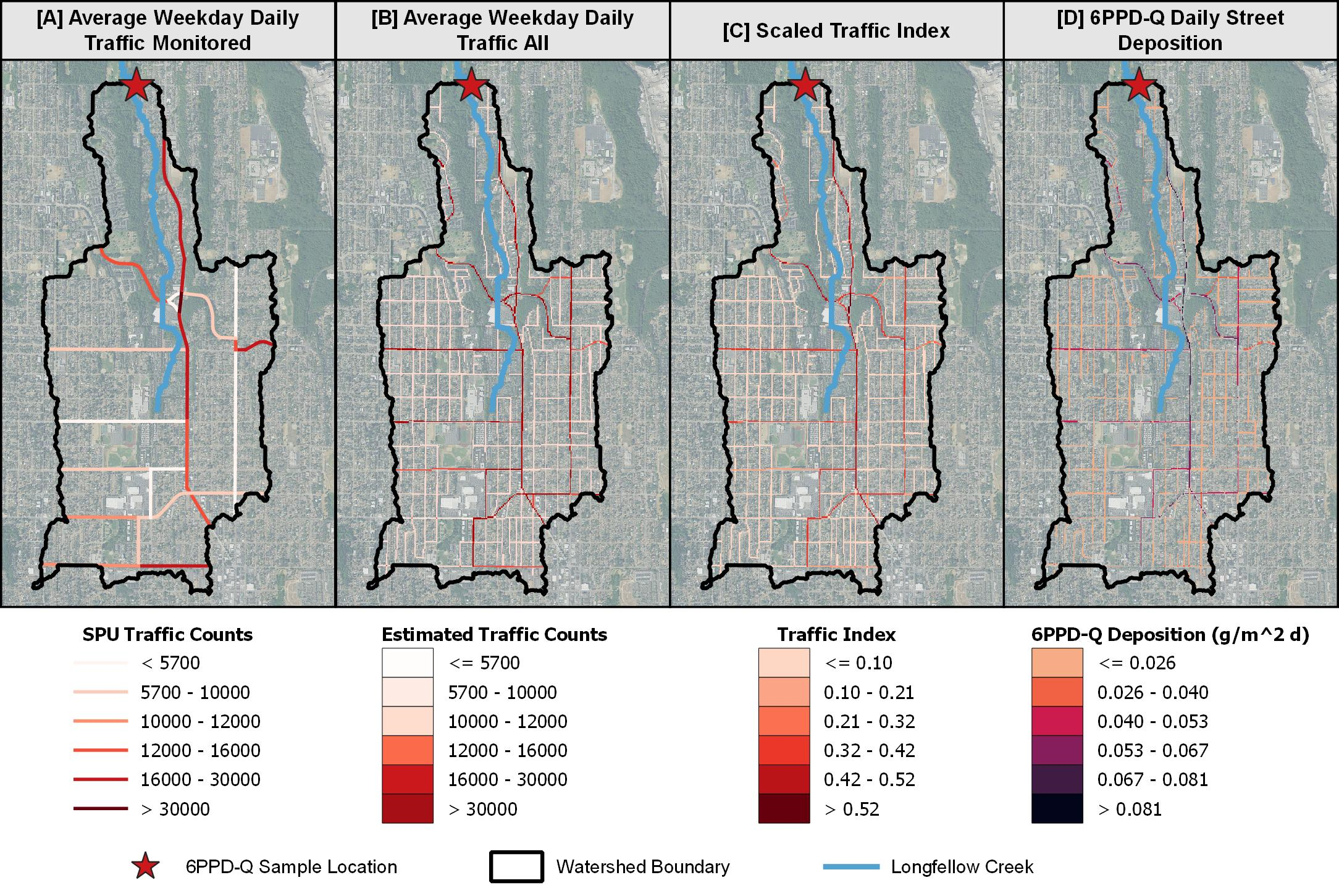

Supplement: Supplement1 [file NIHMS1974508-supplement-Supplement1.zip › Image 3.TIFF]
